# Upregulation of PSMA Expression by Enzalutamide in Patients with Advanced mCRPC

**DOI:** 10.3390/cancers14071696

**Published:** 2022-03-26

**Authors:** Florian Rosar, Robert Neher, Caroline Burgard, Johannes Linxweiler, Mathias Schreckenberger, Manuela A. Hoffmann, Mark Bartholomä, Fadi Khreish, Samer Ezziddin

**Affiliations:** 1 Department of Nuclear Medicine, Saarland University, 66421 Homburg, Germany; florian.rosar@uks.eu (F.R.); s8ronehe@stud.uni-saarland.de (R.N.); caroline.burgard@uks.eu (C.B.); mark.bartholomae@uks.eu (M.B.); fadi.khreish@uks.eu (F.K.); 2 Department of Urology, Saarland University, 66421 Homburg, Germany; johannes.linxweiler@uks.eu; 3 Department of Nuclear Medicine, Johannes Gutenberg-University, 55131 Mainz, Germany; mathias.schreckenberger@unimedizin-mainz.de (M.S.); manuela1hoffmann@bmvg.bund.de (M.A.H.)

**Keywords:** prostate cancer, mCRPC, PSMA, enzalutamide, upregulation

## Abstract

**Simple Summary:**

The prostate-specific membrane antigen (PSMA) which shows overexpression on the cell surface of prostate cancer cells, provides a specific target for molecular imaging and radioligand therapy. In this study, we investigated PSMA upregulation by enzalutamide, an established androgen axis drug, in a cohort (*n* = 30) of patients with advanced, metastatic, castration-resistant prostate cancer (mCRPC). Our results show that short-term enzalutamide medication significantly increases PSMA expression in patients with mCRPC. Therefore, enzalutamide may provide a potential enhancer medication for PSMA-targeted radioligand therapy.

**Abstract:**

In this study, we investigated upregulation of prostate-specific membrane antigen (PSMA) by enzalutamide in a cohort (*n* = 30) of patients with advanced metastatic castration-resistant prostate cancer (mCRPC). Patients were examined by [^68^Ga]Ga-PSMA-11 PET/CT pre- and post-enzalutamide medication (mean 13 ± 7 days). Imaging results were compared based on quantification of whole-body PSMA tumor burden: total lesion PSMA (TLP) and normalized TLP values to liver (TLP-LR) and to parotid gland (TLP-PR). In addition, lesion-based analyses were performed. The median (mean) increases in TLP, TLP-LR and TLP-PR after enzalutamide medication were 10.1% (20.2%), 29.5% (34.8%) and 27.6% (24.4%), respectively. These increases were statistically significant (*p* = 0.002, *p* < 0.001, and *p* < 0.001), while prostate-specific antigen (PSA) serum values did not change significantly (*p* = 0.483). The increase was independent of prior patient exposure to enzalutamide. SUV_max_ increased substantially (>10%) in 49.6% of target lesions. The relative change was significantly higher in the subgroup of lesions with SUV_max_ < 10 (*p* < 0.001). In conclusion, short-term enzalutamide medication significantly increases PSMA expression in patients with mCRPC, irrespective of prior enzalutamide exposure. The relative PSMA upregulation effect seems to be more pronounced in lesions with only moderate baseline PSMA expression. Enzalutamide may provide a potential enhancer medication for PSMA-targeted radioligand therapy.

## 1. Introduction

Prostate cancer is one of the most frequent malignancies in men worldwide [1]. While most patients with metastatic prostate cancers initially respond well to androgen-deprivation therapy (ADT), virtually all patients ultimately develop metastatic castration-resistant prostate cancer (mCRPC) during disease course [2,3]. In this mCRPC setting, various treatments have proven to prolong survival, including the following: chemotherapy with docetaxel [4] or cabazitaxel [5]; therapy with new androgen axis drugs (NAAD), such as abiraterone [6] or enzalutamide [7]; therapy with PARP-inhibitors, such as olaparib [8]; and bone-seeking therapy with ^223^Ra [9]. These therapies have different therapeutic mechanisms. For example, enzalutamide targets multiple steps in the signaling pathway of androgen receptor (AR) and inhibits the binding of androgen to AR, the nuclear translocation and DNA binding of androgen–AR complex and the recruitment of coactivators [7,10]. Besides these established therapies, radioligand therapy (RLT), targeting the prostate-specific membrane antigen (PSMA) using PSMA ligands labeled with a beta emitter (e.g., [^177^Lu]Lu-PSMA-617), has shown promising survival data in patients with mCRPC in various retrospective studies [11,12,13], in prospective phase II trials [14,15] and in a recently published phase III trial [16]. PSMA is a type-II transmembrane glycoprotein, which shows overexpression on the cell surface of prostate carcinoma cells [17] providing new ways of targeted molecular imaging (PSMA PET/CT) and radioligand therapy (PSMA-RLT) [18,19]. In principle, intense PSMA expression forms the basis for a potential response to PSMA-RLT. The expression of PSMA, however, varies from patient to patient and even between different metastases, which may impact the outcome of PSMA-RLT [20]. It is known from preclinical research that PSMA expression in prostate cancer cells is inversely modulated by the AR-signaling cascade, eventually involving the FOLH1 gene, which encodes PSMA [21,22]. Some preclinical and clinical studies have shown that PSMA expression can be increased by enzalutamide [23,24,25,26,27,28,29], which we also recently communicated in a small cohort of advanced mCRPC patients [30]. However, more data is needed to support these findings. In this subsequent study, we investigated PSMA upregulation by enzalutamide in a larger cohort of advanced mCRPC patients.

## 2. Methods

### 2.1. Patients and Ethics

Data of thirty patients with mCRPC in preparation for PSMA-RLT were included in this monocenter study and were analyzed retrospectively. All patients received a baseline [^68^Ga]Ga-PSMA-11 PET/CT, followed by oral enzalutamide medication and a second [^68^Ga]Ga-PSMA-11 PET/CT. The mean duration of enzalutamide medication was 13 ± 7 days, starting 7 ± 11 days after baseline PET/CT and continuing until the second PET/CT. The mean time between both PET/CT scans was 20 ± 12 days. Twenty-eight patients received 160 mg and two patients 80 mg due to mild side effects of previous enzalutamide treatment. The mean age of the patients was 69 ± 9 years. All patients were heavily pretreated and had a high tumor burden with bone metastases (27/30), lymph node metastases (22/30) and visceral metastases (6/30). Detailed information about the patients is presented in Table 1.

[^68^Ga]Ga-PSMA-11 PET/CT was performed on a compassionate use basis under the German Pharmaceutical Act §13 (2b). Patients gave written consent after being thoroughly informed about the risks and potential side effects of this examination and enzalutamide medication. Additionally, patients consented to publication of any resulting data in accordance with the Declaration of Helsinki. The retrospective analysis was approved by the local Institutional Review Board (ethics committee permission number 140/17, approved on 13 July 2017). 

### 2.2. Image Acquisition

For PET imaging, a mean activity of 127 ± 18 MBq [^68^Ga]Ga-PSMA-11 was applied, followed by a 500 mL infusion of NaCl 0.9%. In accordance with standard procedures for prostate cancer imaging [31], the time between injection and PET acquisition was 64 ± 9 min. All PET/CT scans were performed using a Biograph mCT 40 PET/CT scanner (Siemens Medical Solutions, Knoxville, TN, USA) ^18^F-accredited by European Association of Nuclear Medicine (EANM) Research Ltd. With respect to ^68^Ga, inhouse phantom measurements were also routinely performed to allow valid PET quantification. The PET acquisition was conducted from vertex to proximal femur with a 3 min acquisition time per bed position (extended field of view: 21.4 cm). CT data was acquired in low-dose technique using an X-ray tube voltage of 120 keV and a modulation of the tube current by applying ‘CARE Dose4D’ with a maximal tube current time product of 30 mAs. The PET data sets were reconstructed using an iterative 3D OSEM (ordered subset expectation maximization) algorithm (3 iterations, 24 subsets) with Gaussian filtering and slice thickness of 5 mm. Random correction, decay correction, scatter attenuation and attenuation correction were applied.

### 2.3. Quantification of PSMA Expression 

**Figure 1:**
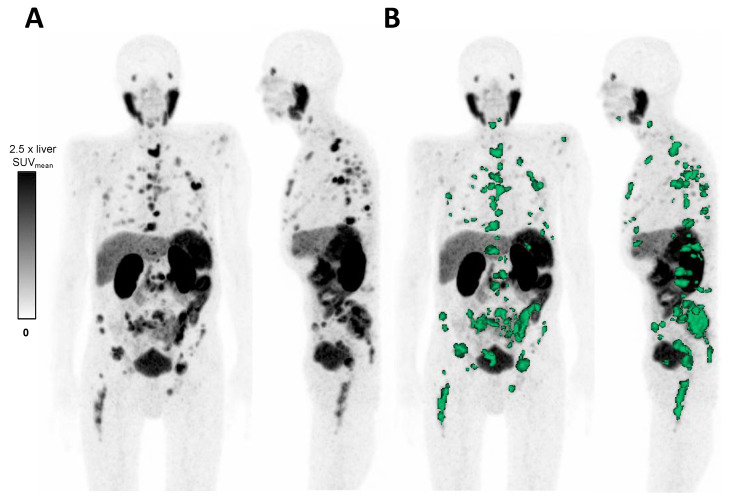
(**A**) MIP (maximum intensity projection) of [^68^Ga]Ga-PSMA-11 PET in an advanced mCRPC patient. (**B**) Semi-automatic tumor segmentation by Syngo.via (Enterprise VB 60, Siemens, Erlangen, Germany). SUV windowing was set from 0 to 2.5 times SUV_mean_ of the liver. The delineated tumor volume is shown in green.

To evaluate PSMA expression, the whole-body total lesion PSMA (TLP), defined as the summed products of SUV_mean_ × PSMA-based tumor volume, was quantified in [^68^Ga]Ga-PSMA-11 PET/CT for each patient pre- and post-enzalutamide medication (patient-based analysis). Quantitative analyses were performed using Syngo.via (Enterprise VB 60, Siemens, Erlangen, Germany) by applying a semi-automatic tumor segmentation algorithm (Figure 1). Following Ferdinandus et al., a standard uptake value (SUV) threshold of 3.0 was set to delineate tumor lesions [32]. Considering the physiological organ uptake in healthy liver tissue (in general SUV > 3.0), a SUV threshold of 1.5 × SUV_mean_ of the liver was applied for intrahepatic metastases [33]. Physiological uptake in organs, e.g., kidneys or salivary glands, were manually excluded. In addition, we normalized TLP values to healthy PSMA-positive tissue as liver and parotid gland: TLP-to-liver ratio (TLP-LR) and TLP-to-parotid gland ratio (TLP-PR). TLP-LR and TLP-PR were calculated as the quotient of TLP and SUV_mean_ of the respective organ. To evaluate the effect of enzalutamide on metastases with different PSMA expression (lesion-based analysis), an independent reader with long experience in molecular imaging (F.K.) determined up to ten bone metastases per patient in baseline [^68^Ga]Ga-PSMA-11 PET/CT (up to five with moderate and up to five with high PSMA expression). All metastases had to be larger than 1 cm in diameter to allow valid PET quantification. SUV_max_ of the identified lesions were measured in baseline and at follow up [^68^Ga]Ga-PSMA-11 PET/CT. 

For statistical analysis Wilcoxon matched pairs signed rank test and Mann–Whitney test were applied using Prism 8 (GraphPad Software, San Diego, CA, USA) to determine significant differences. A *p* value < 0.05 was regarded as statistically significant.

## 3. Results

**Figure 2:**
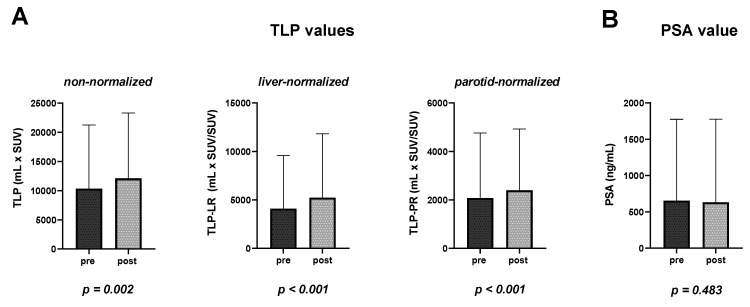
(**A**) Non-normalized total lesion PSMA (TLP), liver-normalized TLP (TLP-LR), parotid-normalized TLP (TLP-PR); (**B**) prostate-specific antigen (PSA) serum levels pre- and post-enzalutamide medication.

**Figure 3:**
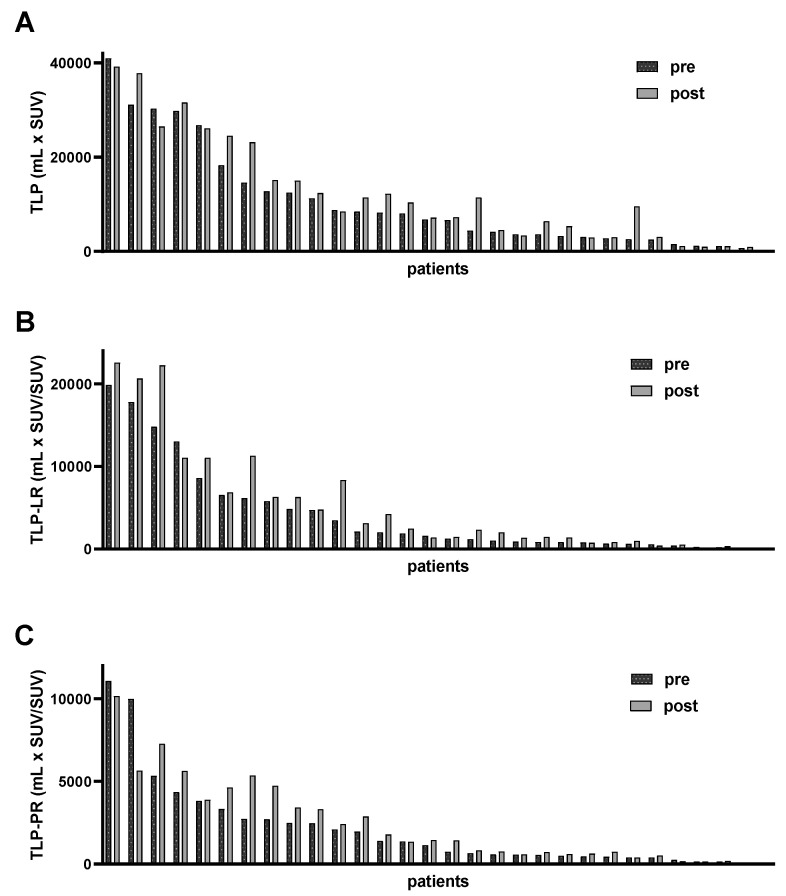
Waterfall plots of individual (**A**) non-normalized total lesion PSMA (TLP), (**B**) liver-normalized TLP (TLP-LR) and (**C**) parotid-normalized TLP (TLP-PR) values pre- and post-enzalutamide medication.

**Figure 4:**
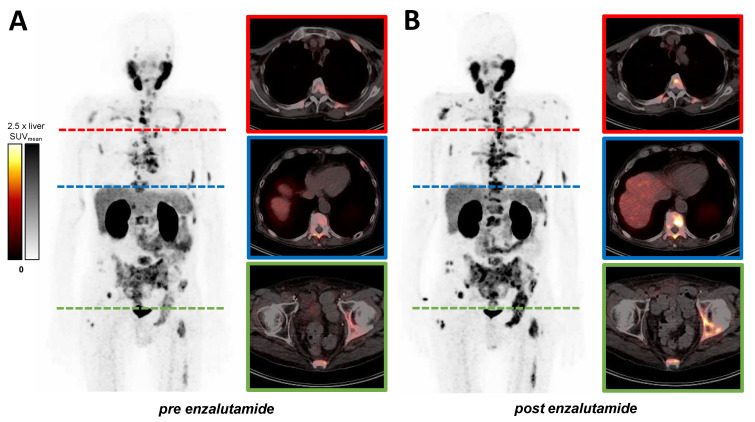
Example of a 68-year-old patient with predominantly bone metastases. Increased uptake can be seen in presented MIP (maximum intensity projection) and exemplary transversal slices of [^68^Ga]Ga-PSMA-11 PET/CT (**A**) baseline (TLP 3611 mL × SUV, TLP-LR 840 mL × SUV/SUV, TLP-PR 455 mL × SUV/SUV, PSA 408 ng/mL) and (**B**) after 18 days of enzalutamide medication (TLP 6440 mL × SUV (+78.3%), TLP-LR 1464 mL × SUV/SUV (+74.3%), TLP-PR 688 mL × SUV/SUV (+38.0%), PSA 189 ng/mL (−53.7%)). SUV windowing was set from 0 to 2.5 times SUV_mean_ of the liver.

In the patient-based analysis, a significantly higher TLP (10,920 ± 10,910 mL × SUV vs. 12,608 ± 11,418 mL × SUV, matched pairs signed rank test *p* = 0.002) was measured after enzalutamide medication (mean 13 ± 7 days) in the follow-up PET/CT examination compared to baseline PET/CT. Consistently, TLP-LR (4096 ± 5464 mL × SUV/SUV vs. 5228 ± 6583 mL × SUV/SUV, *p* < 0.001) and TLP-PR (2072 ± 2690 mL × SUV/SUV vs. 2391 ± 2530 mL × SUV/SUV, *p* < 0.001) values showed a significant increase, whereas prostate-specific antigen (PSA) serum levels (652 ± 1122 ng/mL vs. 631 ± 1145 ng/mL, *p* = 0.483) did not change significantly (Figure 2). Additionally, uptake in reference organs (liver and parotid gland) and in the kidney did not change significantly (*p* = 0.155, *p* = 0.309, *p* = 0.465). The overall tumor burden varied from patient to patient; the individual TLP, TLP-LR and TLP-PR values pre- and post-enzalutamide medication are presented in Figure 3. In concordance with TLP quantification, increased uptake could also be identified visually on [^68^Ga]Ga-PSMA-11 PET/CT images. Figure 4 shows a representative example of increased tracer uptake in a [^68^Ga]Ga-PSMA-11 PET/CT of an mCRPC patient after 18 days of enzalutamide medication, despite a decrease in the serum PSA level. 

The overall median (mean ± standard deviation) increased in non-normalized TLP, TLP-LR and TLP-PR after enzalutamide medication was 10.1% (20.2 ± 36.1%), 29.5% (34.8 ± 43.1%) and 27.6% (24.4 ± 33.4%), respectively.

**Figure 5:**
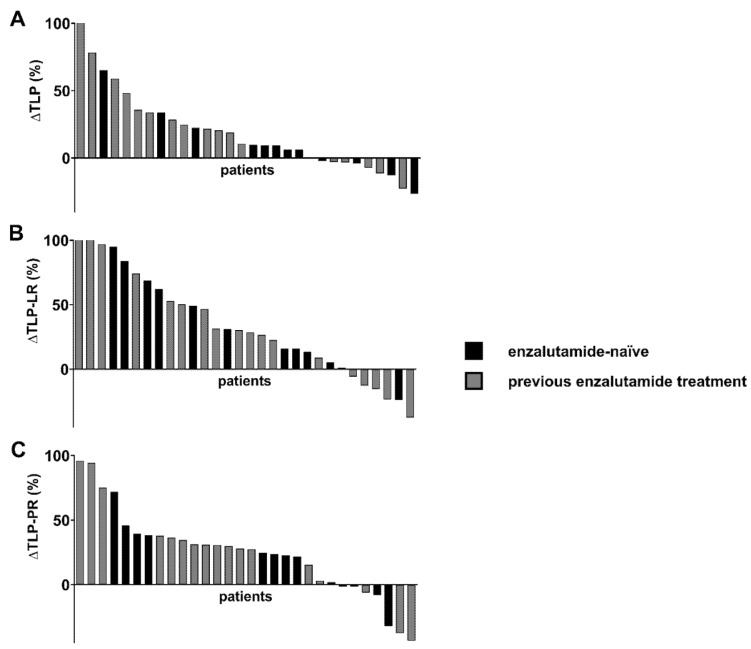
Waterfall plots of individual relative changes in (**A**) non-normalized total lesion PSMA (TLP), (**B**) liver-normalized TLP (TLP-LR) and (**C**) parotid-normalized TLP (TLP-PR) after medication with enzalutamide. Black—enzalutamide-naïve patients. Grey—patients with previous enzalutamide treatment. Values over 100% were cropped for simplification.

In total, 15/30 (50.0%), 21/30 (70.0%) and 21/30 (70.0%) patients showed a substantial increase in TLP, TLP-LR and TLP-PR of more than 10%, respectively. The individual relative changes in TLP, TLP-LR and TLP-PR levels for all patients are shown in Figure 5. Of note, the increment in the subgroup of patients previously treated with enzalutamide was comparable to that in patients without prior enzalutamide therapy for all three parameters—TLP, TLP-LR and TLP-PR (*p* = 0.20, 0.98 and 0.60). In contrast, a substantial decrease in TLP, TLP-LR and TLP-PR was observed in 4/30 (13.3%), 5/30 (16.7%) and 3/30 (10.0%) patients.

**Figure 6:**
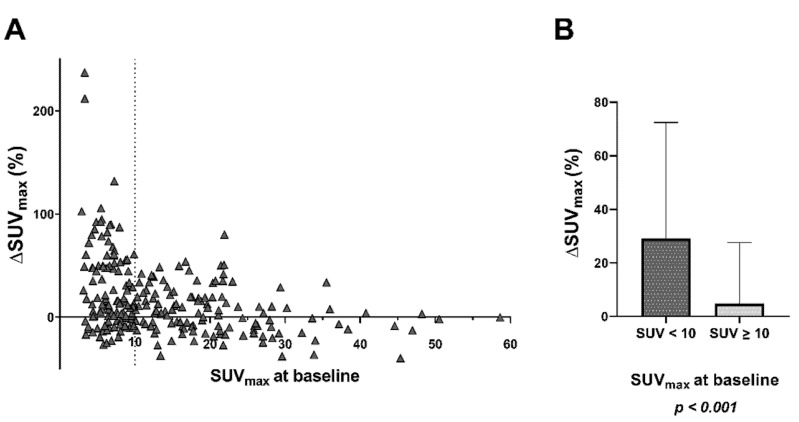
Relative change of SUV_max_ in 242 bone metastases (maximum of 10 per patient) after enzalutamide medication (**A**) for each lesion individually, (**B**) categorized in subgroups with initial SUV_max_ < 10 and SUV_max_ ≥ 10, respectively.

In lesion-based analysis, 242 bone lesions of 27 patients with bone metastases (maximum of 10 per patient) were determined as target lesions and analyzed: 115 with an SUV_max_ < 10 and 127 with an SUV_max_ ≥ 10. The mean SUV_max_ of all lesions was 14.4 ± 10.8 at baseline and 15.5 ± 10.2 after enzalutamide medication. This difference was statistically significant (*p* < 0.001). Lesion-related change in SUV_max_ was highly heterogeneous intra- and inter-individually. In total, 120/242 (49.6%) metastases showed a substantial increase of >10%. Figure 6 presents the relative changes of SUV_max_ for each lesion, as well as categorized in subgroups with SUV_max_ < 10 and SUV_max_ ≥ 10, respectively. The relative change was significantly higher in the subgroup with SUV < 10 (30.4 ± 42.6% vs. 4.8 ± 22.8%, *p* < 0.001). However, in both groups, there were also a quite number of lesions with decreasing uptake.

## 4. Discussion

In this study, we examined the PSMA upregulation by antiandrogen therapy with enzalutamide. After short-term medication with enzalutamide, whole-body total lesion PSMA (TLP) increased significantly, while PSA serum values did not change significantly. This constellation indicates that PSMA expression was significantly increased by enzalutamide medication and the measured increase in PSMA molecules was not caused by disease progression. The increase in TLP was corroborated by normalized TLP values to healthy PSMA-positive tissue as liver and parotid gland (TLP-LR, TLP-PR). Interestingly, PSMA upregulation was highly heterogeneous intra- and inter-individually, with the clinically meaningful observation that lesions with moderate baseline PSMA expression show higher potential upregulation than lesions with high baseline PSMA expression. Some patients showed a substantial decrease in PSMA expression in target lesion or total tumor burden, which might be attributed to therapeutic response to enzalutamide. 

To the best of our knowledge, this is the first study in a larger patient cohort (*n* = 30) showing that antiandrogen therapy with enzalutamide amplifies PSMA expression in mCRPC. The presented data are consistent with our preliminary experience in *n* = 10 patients [30] and other clinical [23,24] and preclinical studies [25,26,27,28,29] on enzalutamide. Emmett et al. observed in *n* = 7 mCRPC patients (6 receiving enzalutamide and 1 abiraterone) that PSMA expression increased significantly after a short time and subsequently plateaued [23]. A variability in PSMA expression after enzalutamide medication was also noted by Aggarwal et al., with an increase in almost half of analyzed target lesions in *n* = 8 patients with prostate cancer (4 castration-resistant and 4 castration-sensitive patients) [24]. In a preclinical setting, Murga et al. and Kranzbühler et al. observed an increased PSMA intensity by enzalutamide in prostate cancer cell lines as LNCaP and C4-2 [25,26]. In analogy, Evans et al. and Lückerath et al. demonstrated increased PSMA expression in murine prostate cancer xenograft models after treatment with enzalutamide [27,28]. Recently, Staniszewska et al. were able to confirm these results in 22Rv1, C4-2 and LNCaP cells as well as in xenograft bearing mice and communicated this effect also in *n* = 1 mCRPC patient receiving enzalutamide [29]. Another similar clinical case was reported by Wondergem et al., who noticed increased PSMA uptake in lymph node metastases after initiation of enzalutamide treatment, whereas lymph node volume and PSA decreased over time [34]. Besides enzalutamide, PSMA upregulation was also described for LHRH analogues and other antiandrogen drugs, as abiraterone or dutasteride [25,26,28,35,36,37,38,39]. In prostate cancer cells, abiraterone and dutasteride showed analogous preclinical PSMA upregulation effects to enzalutamide [25,26].

This effect on PSMA expression should be considered when interpreting PSMA imaging after initiation or modification of hormonal treatment to avoid false interpretation [23]. For example, when evaluating response by molecular imaging, the phenomenon of upregulation should be taken into account when biochemical response is stable or regressive, but uptake increases in PSMA PET/CT.

More importantly, this effect on PSMA expression potentially has also important implications on PSMA-RLT. Our results strongly suggest that enzalutamide can be used to upregulate PSMA expression and thus amplify the antitumor mechanism of PSMA-RLT. Interestingly, in our study the effect of upregulation in the subgroup of patients who were previously treated with enzalutamide was comparable to that in enzalutamide-naïve patients. This is in line with our preliminary experience [30] and offers the possibility to use enzalutamide in combination with PSMA-RLT independent of prior enzalutamide treatment. For this reason, enzalutamide may provide a potential enhancing medication for PSMA-RLT, which has also been suggested by other groups [23,25,26]. With regard to the observation that lesions with only moderate baseline PSMA expression (such as SUV_max_ < 10) show higher potential upregulation than lesions with high baseline PSMA expression, the approach of applying enzalutamide for aiming at induction of higher PSMA expression may particularly motivating in patients with low-uptake patterns of mCRPC. Additionally, patients with inadequate response to PSMA-RLT might benefit from co-medication with enzalutamide. Moreover, the combination of both therapeutics may also provide a synergistic antitumor effect regardless of any PSMA upregulation phenomenon, especially in enzalutamide-naïve patients. Furthermore, it should be examined whether enzalutamide can be used to recruit patients for PSMA-RLT who were previously ineligible due to insufficient PSMA expression [29]. These approaches should be evaluated in larger studies, ideally in prospective clinical trials. 

## 5. Limitations

The study has several limitations. Most importantly, the data are based on a retrospective monocenter study with a limited number of patients, which precluded further analyses to systematically determine the required time and dose of enzalutamide. In particular, the varying duration of enzalutamide medication and single follow-up PET/CT setting limits further deductions. In addition, lesion-based analysis was only performed for bone metastases as the majority of patients had dominant osseous tumor burden. Analyses and comparisons of other sites of metastases would also be of interest and should be performed in a larger cohort.

## 6. Conclusions

Our results show that short-term enzalutamide medication significantly increases tumoral PSMA expression in patients with metastatic castration-resistant prostate cancer, irrespective of prior exposure to the drug. The relative PSMA upregulation effect seems to be more pronounced in lesions with only moderate baseline expression of the target. Enzalutamide may offer potential augmentation of PSMA-RLT by enhancing the antitumor mechanism, certainly deserving evaluation as synergistic RLT co-medication in a prospective setting. 

## Figures and Tables

**Table 1:** Characteristics of the patient cohort.

Characteristics	Mean ± SD/*n* (%)
Age (years)	69 ± 9
Enzalutamide medication	
Dose	
160 mg/day	28 (93.3%)
80 mg/day	2 (6.7%)
Duration (days)	13 ± 7
Prior treatments	
Prostatectomy	11 (36.7%)
Radiation	7 (23.3%)
ADT	30 (100%)
NAAD	26 (86.7%)
Enzalutamide	18 (60.0%)
Abiraterone	21 (70.0%)
Enzalutamide and Abiraterone	13 (43.4%)
Chemotherapy	26 (86.7%)
Docetaxel	26 (86.7%)
Cabazitaxel	14 (46.7%)
Docetaxel and Cabazitaxel	14 (46.7%)
^223^Ra therapy	2 (6.7%)
Sites of metastases	
Bone	27 (90.0%)
Lymph node	22 (73.4%)
Visceral	6 (20.0%)

ADT—androgen-deprivation therapy; NAAD—new androgen axis drugs; SD—standard deviation.

## Data Availability

The datasets used and analyzed during the current study are available from the corresponding author on reasonable request.
